# Effects of climate change on pastoral households in the Harshin District of the Somali Region, Ethiopia

**DOI:** 10.4102/jamba.v14i1.1202

**Published:** 2022-07-27

**Authors:** Tigist Abrham, Muluken Mekuyie

**Affiliations:** 1 Department of Natural Resource Management, College of Dryland Agriculture, Jijiga University, Jijiga, Ethiopia; 2 Wondo Genet College of Forestry and Natural Resource, Hawassa University, Hawassa, Ethiopia

**Keywords:** pastoralist, agropastoralists, climate variability, adaptation strategies, households

## Abstract

This study was conducted in the Harshin District of the Somali Region, Ethiopia, to understand the climate change trends, their consistency with pastoralists’ perceptions and their effects on pastoral households. The study used both qualitative and quantitative data collected from 143 households through household surveys. Focus group discussions and key informant interviews were also employed to triangulate and substantiate the reports from household surveys. Data were analysed using Statistical Package for Social Sciences (SPSS) and chi-square tests to test a degree of significance between the pastoral and agropastoral households for the impact of climate change. Mann–Kendall’s trend test and Sen’s slope estimator were employed to determine climate change trends of the study area. The result showed that pastoral households perceived an increasing trend in annual temperature and a decreasing trend in annual and seasonal rainfall. Mann–Kendall’s trend analysis confirmed pastoral communities’ perceptions of higher temperatures and rainfall variability, with the exception of a long-term decline in rainfall. The findings further indicated that six droughts (one severe and five moderate) were observed for the period 1983–2017. The result indicated that the significant increase in temperature along with high interannual and seasonal rainfall variability have been causing adverse impacts on crop and livestock production. Therefore, there is a need to provide drought-tolerant and early-maturing crops and improved livestock breeds for pastoral households. Water-related interventions such as small-scale irrigation farming and water harvesting during good rainy seasons is also paramount to enhance climate resilience of the local people.

## Introduction

By 2100, the Intergovernmental Panel on Climate Change (IPCC) anticipates a 1.1 °C – 6.4 °C increase in global mean temperature, resulting in storms and floods as well as a rise in sea level as a result of ocean thermal expansion and ice sheet and glacier melting (IPCC 2014). According to climate change projections, the significant negative effects of climate change would increasingly hurt many underprivileged countries around the world, owing to their limited adaptation potential (Ajuang et al. 2016). Ethiopia is vulnerable to the effects of climate change amongst sub-Saharan African countries because it lacks access to adaptive capacity-building components, such as information, resources and technology, and because of its dependency on rain-fed agriculture (Asmare, Teklewold & Mekonnen 2019; Mekonnen, Woldeamanuel & Kassa 2019).

Climate change consequences on rural smallholder farmers are very sensitive because of recurring declines in agricultural yields, loss of livelihood assets and opportunities (IPCC 2007; Thornton et al. 2014). Pastoral and agropastoral groups of Ethiopia, which account for 60% of the total land area and 12% of the population, raise livestock in drought-prone areas (Samuel 2016). Thus, the recurrent drought is linked to the decline in livestock population and production in these areas (Berhe et al. 2017). Despite the difficulties, farming has continued in these regions for many years, with farmers relying on their indigenous expertise to sustain their livelihoods (Charles, Kalanda & Ngongondo 2014).

Rainfall in Ethiopia is very variable in terms of amount and distribution between regions and seasons, making long-term rainfall trends difficult to predict (World Bank Group 2020). When averaged throughout the entire country, studies show that rainfall remained relatively consistent; nevertheless, a falling tendency has been detected in northern and south-western Ethiopia (Zerga and Gebeyehu 2016). Ethiopia’s yearly average temperature has risen by 1 °C since 1960, at a pace of 0.25 °C each decade (World Bank Group 2020). In Ethiopia, topography, which has huge regional variances throughout its major physiographic areas, has a significant impact on rainfall variability (Zerga and Gebeyehu 2016). However, there are few researches on climate features at regional and local levels (Girmay, Gebreselassie & Bajigo 2018). Hence, there are few studies spanning highland and lowland regions that can help us identify local-level climatic change trends.

The Somali regional state is one of Ethiopia’s most pastoral and agropastoral regions. The federal and regional governments, as well as humanitarian organisations, are concerned about the region’s repeated drought and chronic food shortages (Girmay et al. 2018). The region’s pastoral and agropastoral livelihood systems are sensitive to the negative effects of climate change (Fratkin 2014), as they rely on basic natural resources like water and pastures to survive. For generations, pastoral and agropastoral households have been adapting to climatic change. However, pastoralists have recently become less adaptable to climate change and variability as a result of increasing tendencies towards repeated droughts, high geographical and temporal rainfall variability and existing poor socioeconomic features (Ayal & Leal Filho 2017).

Whilst studies about trends of climate variability and its impact on farmers’ livelihoods are abundant in highland areas of Ethiopia, where there is a relatively enabling environment for communities to respond better to the impacts of climate change (Aklilu & Alebachew 2009; Tadese & Alemayehu 2017), few studies have been conducted in marginal areas of pastoral communities (Debela et al. 2015; Deressa, Hassan & Claudia 2008; Lemma et al. 2013). According to the findings of a study conducted by Deressa et al. (2008), the Somali national regional state is one of the country’s two most vulnerable regions to climate change impacts. The authors have highlighted the need for more research at the local and district levels as their study comes to a close. As a result, understanding climate change trends and their impact on pastoralists’ livelihoods at a local level is critical for finding adaptation and mitigation options. Climate-induced shocks are expected to be more severe in pastoral communities, as the environment is fragile and people’s adaptive capacity is low. However, studies on the impact of climate change and variability on pastoral communities are very limited, which is particularly true at the local level of analysis. Therefore, the goal of this study was to investigate climate change trends, their consistency with pastoralists’ perceptions and their effects on pastoral households in the Harshin District of the Somali Region, Ethiopia.

## Materials and methods

### The study area

**FIGURE 1:**
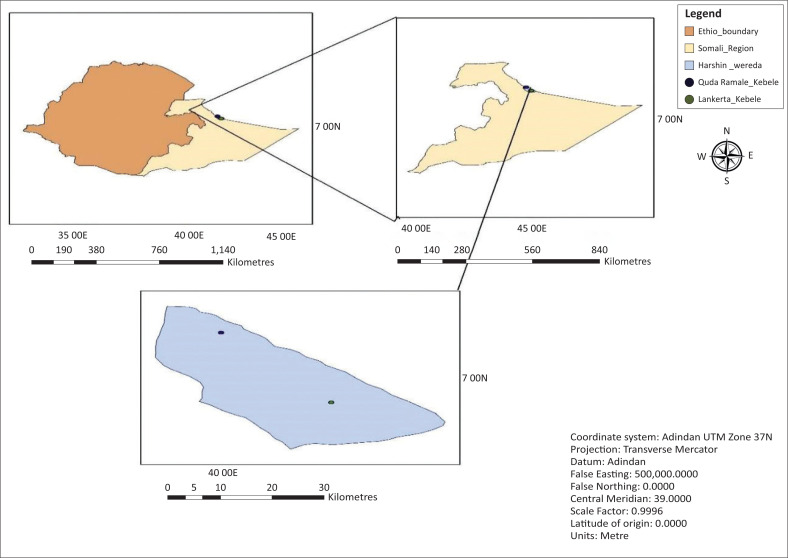
Map of the study area.

Despite periodic variations in climate change consequences, the severity of these impacts differs throughout Ethiopian regions. The climate change impacts in the Somali Region, which includes semi-arid to arid agroecology, are highly diverse (Lemma et al. 2013). The regional communities’ resilience to climate change has been compromised as a result of repeated shocks and pressures (Lemma et al. 2013). Drought is a continuous aspect of the region’s varied climate and weather extremes, with an inexorable effect (Michael 2017). Despite the fact that drought is one of the most painful natural disasters in arid tropical environments, the socio-economic consequences are severe in districts with less than 500 mm of annual precipitation (Asrat & Simane 2018). The Harshin District was chosen to represent the arid and semi-arid pastoral and agropastoral livelihood zones in the Somali Region of Ethiopia using a purposeful random sampling approach. Harshin is located 730 km away from the capital city of Ethiopia, Addis Ababa. The district covers a total area of 5120.12 km^2^ and borders Kebribeyah District in the west, Somaliland to the north and east, and Aware and Degehabur Districts to the east and south, respectively. The district is geographically located between longitude 9° and 21° in the north, 42° and 48° in the east, 29° and 35° in the west and 42° and 53° in the south-east (Figure 1). Its altitude ranges from 1302 m to 1700 m above sea level. The climate is generally hot and dry with a mean annual temperature ranging from 28 °C to 36 °C. The annual rainfall ranges from 300 mm to 400 mm with limited and unreliable rainfall with two peak rainy seasons of *Gu* (April–May) and *Deyer* (October–December). The *Gu* rains are the most important for pastoralists. The Harshin District’s location is on the boundary between areas in the Somali Region that receive *Karan* rains from late July to September (Berhanu, Melesse & Seleshi 2013).

### Research design, sampling approach and sample size determination

To address the study’s desired objectives, a mixed research approach was adopted. A multistage sampling procedure was used to determine the appropriate sample size and research units. Firstly, the Harshin District was purposefully chosen to exemplify the district most vulnerable to climate risks and anomalies in Somalia’s arid and semi-arid pastoral and agropastoral zones. We intended to investigate climate change effects on these two farming systems (pastoralists and agropastoralists) because they could be affected differently. Secondly, two villages, namely Lankerta and Qudha Ramalle from pastoral and agropastoral villages, respectively, were randomly selected out of 11 villages in the district. Around 143 sample households were selected using Yemane’s (1967) statistical technique. The formula is presented as follows:


$$n=\frac{N}{1+N(e)2}$$ [Eqn 1]

where *n* is the sample size, *N* is the population size (total household heads size) and *e* is the level of precision.

Random sampling was employed to select respondents from household lists in each village, whilst probability proportional to size was utilised to determine the number of respondents in each village. Participants in focus groups and key informant interviews (KIIs) were chosen using a purposeful random sample technique based on their interest and notoriety in the area of study.

### Data collection

The study used a mixed-methods approach to collect quantitative and qualitative data in order to identify cases with a lot of information and capture different points of view (Creswell & Plano Clark 2018). The necessary data were gathered using both primary and secondary data sources. The primary data were collected using an in-depth interview schedule to learn about the household perceptions of climate change and perceived impacts on the pastoral communities. A household survey was undertaken at the village level from November to May 2019 by four enumerators. Prior to data collection, a semistructured interview schedule was developed and pretested to assess the questionnaire’s consistency, logical flow and interquestion linkages. This contributed to the refinement of questions that were determined to be confusing or irrelevant, as well as the verification of the content’s rationale and dependability. As a result, the questionnaire was divided into three parts: basic household information, perception of climate change and perceived effects of climate change on pastoralists.

In-depth perception data were obtained through focus group discussions (FGDs) and KIIs to analyse the retrospective information concerning climate change trends and their impacts. Because members would act as a check for one another, both are accurate devices for acquiring unbiased and nonexaggerated qualitative data from discussants (Abrha & Simhadri 2015). Elders, women and youth groups participated in different FGDs in each *kebele* [ward]. Each group had 8–12 participants in it. To get comprehensive and diverse climatic knowledge and associated effects, KIIs were conducted with well-versed community members and concerned professionals at various levels. The discussion took place in the native language and was recorded for later translation into English. The secondary data were collected from journals and unpublished data from both regional and district offices such as the Agricultural and Rural Development Bureau (ARDB) and the Productive Safety Net Program (PSNP).

The Ethiopian National Meteorological Agency (NMA) provided 35 years of temperature and rainfall data (1983–2017) to compare and validate the farmers’ perceptions with actual meteorological trends and come up with reasonable recommendations.

### Data analysis

Collected qualitative and quantitative data were checked, corrected, coded and encoded in a computer and then analysed to extract meaningful information. Different data analysis techniques were employed as both qualitative and quantitative data were collected. The qualitative data that were obtained through KIIs and FGDs were narrated and summarised. Descriptive statistics such as frequency, percentage and mean were employed to determine and assess respondents’ socioeconomic characteristics and perceived impacts of climate variability through the Statistical Package for Social Sciences (SPSS) version 22 software. Independent sample *t*-test and chi-square test were also employed to test the existence of a significant difference between agropastoralists and pastoralists with respect to the livestock owned, family size and perceived level of climate change impact.

### Climate change and variability trend analysis

Long-term trends of the seasonal and annual rainfall were determined using the nonparametric Mann–Kendall’s test and Sen’s slope estimator. Coefficient of variation (CV) was calculated as the ratio of standard deviation to the mean following Hare (1983) to identify the temporal variability of rainfall during the observation period. According to Hare (1983), CV is used to classify the degree of variability of rainfall as less (*CV* < 20%), moderate (*CV* ranges from 20% to 30%) and highly variable (*CV* greater than 30%). Moreover, the precipitation concentration index (PCI), as suggested by Oliver (1980), is a robust indicator of the temporal distribution of rainfall. The equation to calculate PCI at an annual scale is described as follows:


$$PCI_{Annual}=\left[\sum_{i=1}^{12}p_{i}^{2}/{\left(\sum_{i}^{12}p_{i}\right)}^{2}\right]\times 100$$ [Eqn 2]

where

*Pi* = the rainfall amount of the *i*th month and

Σ = summation over the 12 months

The PCI was also analysed for seasonal rainfall using the following equation:


$$PCI_{seasonal}=\left[\sum_{i=1}^{3}p_{i}^{2}/{\left(\sum_{i}^{3}p_{i}\right)}^{2}\right]\times 25$$ [Eqn 3]

According to Oliver (1980), PCI values of less than 10 indicate uniform monthly distribution of rainfall, values between 11 and 15 denote irregular rainfall distribution, values from 16 to 20 indicate highly irregular rainfall distribution and values above 21 indicate very high irregular distribution.

Drought severity classification was computed using the standardised rainfall anomalies (SRA) method in the study area over the period of observation, which is described as


SRA=(Pi−Pm/ð) [Eqn 4]

where SRA is standardised rainfall anomaly, *Pi* is annual rainfall in year *i, Pm* is long-term mean annual rainfall over a period of observation and *ð* is standard deviation of annual rainfall over the period of observation. The drought severity classes are extreme drought (SRA < −1.65), severe drought (−1.28 > SRA > −1.65), moderate drought (−0.84 > SRA > −1.28) and no drought (SRA > −0.84) (Agnew & Chappel 1999).

### Ethical considerations

This article followed all ethical standards for research without direct contact with human or animal subjects.

## Result and discussion

### Socio-economic characteristics of the respondents

A total of 143 sample households had been interviewed via the questionnaire survey. The results indicated that 73.4% were male and 26.6% were female. In addition, 87.4% of households were married. Regarding the educational status, about 83.9% were illiterate. The low level of education suggested that most of the pastoral households in the study area could not find decent employment because of their low level of education. This is similar to Arragaw and Woldeamlak (2017) in the central highlands of Ethiopia, who indicated that the low level of educational status of a household has implications on the adoption of coping and adaptation strategies to climate change and variability. A majority of respondents (88.8%) were engaged in on-farm activities such as sale of crops, livestock and livestock products, whilst 9.1% of the households engaged in off-farm activities such as petty trade, charcoal making, employment and cash-for-work programmes. It was observed that the mean age of the sampled households was 54.63 ± 11.47 and that of agropastoralists and pastoralists was 54.89 and 54.43, respectively. All households were within the active working age group and had relatively long years of farming experience to notice environmental changes.

Regarding the family size of households, the results showed that the mean family size of both pastoral and agropastoral household heads was 5.61 (± 1.81), which is above the national average (5.1). The mean family sizes of agropastoral and pastoral households were 6.22 and 5.13, respectively. The result of the independent *t*-test indicates that there is significant variation between agropastoral and pastoral households in the family size (*t*-test; *t* = 3.767 and *p* < 0.01).

Livestock play a central role in determining the wealth and social status of pastoralists and agropastoralists. The animals reared in the study area predominantly included camels, cattle, sheep, goats, donkeys and chickens. The livestock holdings of each household were calculated in terms of tropical livestock unit (TLU) as recommended by Gryseels (1988) and Shiferaw (1991) for local and crossbreed animals, respectively. The mean livestock size of households was 11.85 TLU (± 6.46). The result indicates that average livestock size of agropastoral households (8.55 TLU) was relatively less than that of the pastoralists (13.19 TLU). The result of the independent *t*-test (*t*-test; *t* = −6.427 and *p* < 0.01) indicates that there was significant variation in livestock size between agropastoral and pastoral households (Table 1). This variation was because of the fact that livestock are a primary source of income in pastoral systems and the latter integrate cultivation as an additional economic activity to improve household income. This result is also in agreement with Nega et al. (2019), who reported that the average livestock holding is relatively larger for pastoral households than agropastorals in Borana, southern Ethiopia.

**TABLE 1:** Socioeconomic characteristics of respondents in continuous variables.

Household characteristics	Agropastoral households		Pastoral households		Overall		*t*	Significance
	Mean	± Standard deviation	Mean	± Standard deviation	Mean	± Standard deviation		
Family size	6.2	± 1.9	5.1	± 1.6	5.61	± 1.8	3.767	0.00†
Age (year)	54.89	± 11.3	54.43	± 11.7	54.63	± 11.5	0.239	0.811
Livestock size (TLU)	8.5	± 3.1	13.2	± 5.0	11.85	± 6.5	−6.427	0.00†
Household income (EB)	17771.4	± 9222.2	11812.5	± 7114	14437.8	± 8608	4.363	0.00†
Land size (ha)	1.8	± 0.7	1.6	± 0.8	1.7	± 0.7	1.607	0.110

*Source:* Field Survey (2019)

† , Significance difference at 1% level of probability.

The findings indicated that the overall mean annual income of the households was 14437.8 ± 8608 Ethiopian Birr (EB). The mean income of agropastoralists and pastoralists was 17771.4 ± 9222.2 and 11812.5 ± 7114.1 EB, respectively. The result of independent *t*-test (*t*-test; *t* = 4.363 and *p* < 0.01) indicated that there was statistically significant variation between the agropastoralists and pastoralists in their annual income, because of the fact that agropastoralists who integrate livestock with cultivation are expected to earn more from diversified income sources.

The household survey result revealed that the overall mean farmland size was 1.7 ± 0.7 ha. Average land size of agropastoral and pastoral households was 1.8 ± 0.7 ha and 1.6 ± 0.8 ha, respectively. The independent sample *t*-test result showed that there was no significant variation between the two production systems in their land size (*t*-test; *t* = 1.607 and *p* > 0.05).

Access to reliable information about seasonal forecasts of the weather conditions and climate variability is necessary to predict the coming weather conditions and to take measures in order to reduce livelihood loss. The result indicated that majority of the respondents from both production systems (92.3%) had no access to climate information.

### Pastoralists’ perceptions of climate change

#### Perceived rainfall trends

It is critical to understand how households perceive temperature and rainfall changes in order to uncover locally available climate variability and adaptation options. The majority of key informant interviewers and FGD participants recognised that rainfall amount, timing and distribution in the study area had changed and varied over the 35-year period investigated. Farmers also stated that the length of the rainy season had changed. According to the findings, 95.8% of respondents noticed a drop in the number of rainy seasons, with late onset and early cessation (Table 2). Furthermore, 23.8% said the seasonal rainfall distribution was erratic. As a result, rainfall unpredictability could have serious effects for pastoralists’ livelihood. Various researches conducted in different locations of Ethiopia have yielded the same conclusion (Bewket 2012; Debela et al. 2019; Tagel & Van Der Veen 2013). Participants in the FGDs agreed that today’s interannual rainfall variability was high and that the start and duration of the rainy season had become unpredictable, making cropping and pastoral activities more difficult to plan and worsening the area’s already severe feed and water shortage.

**TABLE 2:** Perception of the respondent on annual and seasonal rainfall trends.

Perceived trends of rainfall	Percentage of respondents by production system		Total (143)
	Agropastoral (*n* = 63) (%)	Pastoral (*n* = 80) (%)	
No change	**1.6**	**0**	0.7
Increasing	**3.2**	**1.25**	2.1
Decreasing	93.6	97.5	95.8
Don’t know	1.6	1.25	1.4

*Source:* Field Survey (2019)

#### Perceived trends of temperature

In terms of temperature, 94.4% said that the study area’s temperature had risen (Table 3). Other studies covering different sections of Ethiopia (Deressa, Hassan & Claudia 2011; Berhanu & Beyene 2014) found that most Ethiopian farmers are aware that temperatures are rising.

**TABLE 3:** Perception of respondents on trends of temperature in the study area.

Perceived trends of temperature	Percentage of respondents by production system		Total (143)
	Agropastoral (*n* = 63) (%)	Pastoral (*n* = 80) (%)	
No change	1.6	2.5	2.1
Increasing	95.2	93.75	94.4
Decreasing	3.2	2.5	2.8
Don’t know	0	1.25	0.7

*Source:* Field Survey (2019)

#### Perception of pastoral households on drought occurrence

**FIGURE 2:**
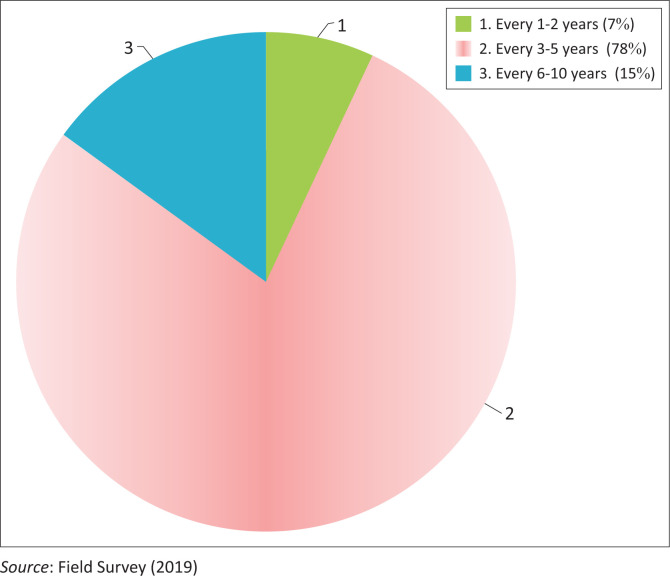
Perception of respondents on drought frequency.

Droughts have afflicted the district and the region in general at various times throughout history, and it is not a new occurrence for pastoral communities. The rain is likely to be more erratic than before, and drought periods are likely to occur more frequently now than before. Drought incidence was examined during the past 35 years, and the majority of respondents from both production systems thought that the drought incidence had become more frequent. Figure 2 shows that 78% of respondents reported that droughts occurred every 3 to 5 years, but 15% of households said that droughts only occur every 1 to 2 years. Furthermore, 7% of respondents stated that droughts occur every 6 to 10 years. According to comparable studies, drought is having an increasingly negative impact on the livelihoods of pastoral and agropastoral communities in eastern Ethiopia (Berhanu & Beyene 2014; Belay, Beyene & Manig 2015). Similarly, FGDs and key informants were asked about the situation of drought occurrence for the last three decades in the study area, and most of them indicated that high frequency of drought was experienced in recent years, which was not familiar before, and it has negative effects on the livelihoods of pastoralists and agropastoralists in the study area.

### Climate change trends

#### Annual rainfall trends

The findings revealed that rainfall in the district has a significant interannual variability (CV = 44.7%), suggesting that high rainfall variability could have a negative influence on livestock and crop productivity. The results of the evaluated meteorological data corroborated the respondents’ perceptions of rainfall variability. The result was supported by Tagel and Van Der Veen (2013), who reported that the increasing year-to-year variability of climate lowers agricultural production with corresponding negative effects on food security in the Tigray Region, northern Ethiopia. Moreover, the results indicated very high irregular distribution of annual rainfall (PCI = 21.4%) (Table 4). The present finding is supported by Fenta (2017), who indicated very high irregular distribution of seasonal and annual rainfall in the southern Afar Region.

**TABLE 4:** Descriptive statistics of annual rainfall in Harshin, 1983–2017.

Observations	Mean (mm)	SD (mm)	Minimum (mm)	Maximum (mm)	CV (%)	Mean PCI
Annual rainfall	406.2	181.8	137	1159	44.7	21.4

*Source:* Field Survey (2019)

SD, standard deviation; CV, co-efficient of variation; PCI, precipitation concentration index.

The long-term annual rainfall had no significant changes for the observed period in the study area (Table 5). The perception of diminishing rainfall trends amongst respondents did not match the actual rainfall trends. This widely perceived decrease in rainfall amount could be because of a decrease in the number of rainy days over the long rainy season or because of higher evapotranspiration and greater moisture stress as a result of the rising temperatures. The result is consistent with Degefu and Bewket (2014) and Solomon et al. (2007), who reported nonsignificant trends in annual rainfall in the Omo-Gibe River Basin and at Jigjiga and Gode stations from 1997 to 2015, respectively.

**TABLE 5:** Trend of annual (mm) rainfall in Harshin from 1983 to 2017.

Mann–Kendall trend test				Sen’s slope estimate
Observations	Start year	End year	Z	Q
Annual	1983	2017	1.4 Ns	0.43

*Source:* Field Survey (2019)

Ns, nonsignificant.

#### Seasonal rainfall trends

In the study area, the *Gu* rainfall, which occurs from April to June, contributed the most to annual rainfall (46.4%) followed by *Karan* rainfall, which occurs from July to September, with a contribution of 28.8% to the total annual rainfall. Rainfall was lowest in *Deyr* season (15.5%), which starts in the month of October and ends in the month of December. The results indicated that the intraseasonal variability was very high for all rainfall seasons (Table 6), which could have an adverse effect on pastoral livelihood by affecting seasonal forage and water availability for their livestock and crop farming. The results of the analysed meteorological data corroborated the respondents’ perceptions of rainfall variability. This study corroborates the findings of recurring rainfall variability in Ethiopia’s tropical and subtropical regions by Viste, Diriba and Sorteberg (2013) and Arragaw and Woldeamlak (2017). Ajuang et al. (2016) detected significant interannual variability in precipitation over the past four decades in Kenya’s Upper Nyakach Division in equatorial eastern Africa.

**TABLE 6:** Descriptive statistics of seasonal rainfall variability.

Observations	Mean (mm)	SD (mm)	Minimum (mm)	Maximum (mm)	CV (%)	Mean PCI
*Gu* (April–June)	190.7	77.2	13.1	379.1	40.5	12.7
*Deyr* (October–December)	64.3	57.98	0	262.1	90.2	17.7
*Karan* (July–September)	116.8	78.3	31	384.6	67.04	14.1

*Source*: Data calculated from NMA reported data, 2019/20

SD, standard deviation; CV, co-efficient of variation; PCI, precipitation concentration index.

The results of the Mann–Kendall statistical tests showed that there were nonsignificant trends during the observation period across all seasons of rainfall (Table 7). Actual seasonal rainfall trends did not match respondents’ perceptions of dwindling rainfall. The result is supported by Gbetibouo (2009), who reported a nonsignificant rainfall trend in large parts of Ethiopia. Similarly, the finding was supported by Yilma and Ulrich (2004), which stated no trend in the annual *Kiremt* and *Belg* rainfall totals and rainy days over central, northern and north-western Ethiopia in the period 1965–2002. The results from analysis of meteorological data imply that intraseasonal and annual rainfall variability and irregular distribution of rainfall across all seasons and years could have a disastrous effect on pastoral livelihoods.

**TABLE 7:** Seasonal rainfall (mm) trends in Harshin 1983–2017.

Mann–Kendall trend test				Sen’s slope estimate
Season	Start year	End year	Z	Q
*Gu* (April–June)	1983	2017	1.02 Ns	1.39
*Karan* (July–September)	1983	2017	1.51 Ns	1.43
*Deyr* (October–December)	1983	2017	1.39 Ns	0.8

*Source*: Data calculated from NMA reported data, 2019/20

Ns, nonsignificant.

**FIGURE 3:**
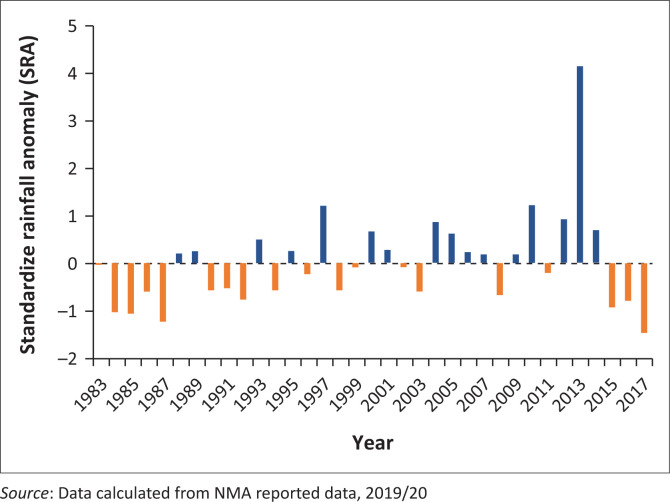
Anomaly of annual rainfall at Harshin from 1983 to 2017.

The standardised anomalies of annual rainfall during the period 1983–2017 showed drier years (53% negative anomalies) (Figure 3). Rainfall with positive anomaly index values indicate above-average rainfall, whereas negative values indicate dry years with varied degrees of intensity. The results indicated that six droughts occurred in the study area from 1983 to 2017, in which one of them was a severe drought which occurred in 2017, whilst the other five were moderate droughts (1984, 1985, 1987, 2015 and 2016).

#### Annual temperature trends

Temperature is another important climate variable that influences the climate of an area. The descriptive statistics, trend and variability of time series maximum, minimum and mean temperature data were analysed on seasonal and annual periods to understand and summarise the long-term temperature of the study area. The mean annual minimum and maximum temperatures between 1983 and 2017 were 14.5 °C (±0.8) and 29.6 °C (±0.5), respectively, with an average of 22.1 °C (Table 8).

**TABLE 8:** Descriptive statistics of annual minimum and maximum temperature.

Temperature	Minimum temperature	Maximum temperature	Average
Lowest	13	28.8	21.3
Highest	16.9	30.7	23.0
Mean	14.5	29.6	22.1
SD	0.8	0.5	0.4

*Source:* Data calculated from NMA reported data, 2019/20

SD, standard deviation.

The Mann–Kendall trend test result indicated that there was a significant increasing trend of annual mean and maximum temperature at a rate of 0.02 °C and 0.026 °C per year, respectively. However, the annual minimum temperature showed a nonsignificant increasing trend at a rate of 0.007 °C per year (Table 9). The actual temperature trends are consistent with the respondents’ perception of rising temperatures. Similar results were reported by Legesse, Ayele and Bewket (2013) and Arragaw and Woldeamlak (2017), who indicated that the annual mean and maximum increased in the central highlands of Ethiopia. The significant increased maximum temperature could stress livestock and the local communities and also cause higher evaporation from soil moisture, which could have adverse effects on crops and pasture growth. Similarly, Solomon et al. (2007) reported that significant increasing temperatures caused water losses via evapotranspiration in the eastern parts of Ethiopia.

**TABLE 9:** Trend of maximum, minimum and mean temperature (°C) in Harshin District (1983–2017).

Mann–Kendall trend test				Sen’s slope estimate
Temperature	Start year	End year	Z	Q
Annual mean temperature	1983	2017	2.88*	0.02
Annual minimum temperature	1983	2017	0.55 ns	0.007
Annual maximum temperature	1983	2017	3.15*	0.026

*Source*: Data calculated from NMA reported data, 2019/20

* , 0.01 significance level; ns, nonsignificant trend at 0.05 significance level.

#### Seasonal temperature trends

The descriptive statistics presented in Table 10 show that the *Gu* season (April–June) was warmer than the other three seasons.

**TABLE 10:** Seasonal mean temperature descriptive statistics (1983–2017).

Season	Minimum	Maximum	Mean	SD
*Gu* (April–June)	22.5	24.1	23.2	0.4
*Karan* (July–September)	21.5	23.4	22.4	0.5
*Deyr* (October–December)	19.8	23.0	21.0	0.8

*Source*: Data calculated from NMA reported data, 2019/20)

SD, standard deviation.

The Mann–Kendall trend test showed that there was a significant increasing trend of mean temperature in all seasons at a rate of 0.02 °C per season for the *Gu* and *Jillal* seasons and 0.03 °C for the *Karan* and *Deyr* seasons (Table 11). The Mann–Kendall trend test for the seasonal minimum temperature indicated significant increasing trends for *Karan* (July–September) and *Deyr* (October–December) seasons at a rate of 0.02 °C and 0.04 °C per year, respectively (Table 11). Similarly, the result for seasonal maximum temperature showed significant increasing trends in *Gu* (April–June) and *Karan* (July–September) seasons by 0.035 °C and 0.037 °C per season, respectively. This increased temperature could impose a challenge on the livelihood of the community, as the area is prone to recurrent drought that can adversely affect the forage and water availability. This result is supported by the reports of NMA (2007) in Ethiopia, which indicated that temperature has been increasing all over the country. Moreover, Hirons et al. (2010) also revealed that in Africa, in all seasons, the median temperature increased and lay between 3 °C and 4 °C, roughly 1.5 times the global. The increasing temperature trend caused higher evaporation of water bodies and soil moisture that had disastrous effects on the seasonal availability of pasture and water for livestock, which impacted the resilience of pastoralists in the Afar Region (Fenta 2017).

**TABLE 11:** Trend of seasonal minimum, maximum and mean temperatures (°C) per season in Harshin (1983–2017).

Minimum temperature (1983–2017)			Maximum temperature (1983–2017)		Mean temperature (1983–2017)	
Season	Test Z	Sen’s slope	Test Z	Sen’s slope	Test Z	Sen’s slope
*Gu* (April–June)	−0.28	0.00	2.34*	0.035	2.10*	0.02
*Karan* (July–September)	2.0*	0.02	2.67**	0.037	3.88***	0.03
*Deyr* (October–December)	1.8+	0.04	1.04ns	0.008	2.07*	0.03
*Jilaal* (January–March)	1.05	0.012	2.27*	0.026	1.69+	0.02

*Source:* Data calculated from NMA reported data, 2019/20

*** , 0.001 significance level;

** , 0.01 significance level;

* , 0.05 significance level; + = 0.1 significance level; ns = nonsignificant trend at 0.05 significance level.

### Perceived impact of climate change and variability

Perception of households on the impact of climate change and variability is presented in Table 12. Pastoral households perceived the top five climate change effects as a decrease in crop yield, a decrease in livestock asset, a lack of water and food and a loss of income. This result corroborates the findings reported by Charles et al. (2014), who indicated that climate variability has induced increased crop failure, higher livestock asset losses, migration, food shortages, biodiversity loss and negative human development effects in the same agro-ecology of southern Malawi. Except for income loss, the χ^2^ test revealed a significant difference in perceptions of climate change impacts between pastoral and agropastoral households (*p* ≥ 0.01).

**TABLE 12:** Perceived impact of climate change and variability in the study area.

Perceived impact	Agropastoral (*n* = 63) (%)	Pastoral (*n* = 80) (%)	χ^2^	*p*
Decline in crop yield	93.7	5	127.54	0.000*
Livestock asset reduction	81	100	16.63	0.00*
Shortage of water	55	80	10.76	0.00*
Food shortage	38	78	31.81	0.00*
Loss of income	81	90	2.398	0.012

*Source*: Field Survey 2019

* Significance at 1% level. Percentages do not add up to 100 because of multiple responses.

#### Impacts of climate variability on crop yield

Local households complained that recurrent droughts, early cessation and late onset of rain, heavy and unseasoned rain and pests have caused crop failure. A majority of agropastoral households (93.7%) have experienced reduced crops because of climate-related shocks and stresses. Droughts and delays in the onset of rains have made the farmlands drier and more difficult to plough and caused stunted growth of crops and slow germination of seeds, resulting in early wilting of the crops and a decline in crop yield (or in some cases, a loss of the entire crop). Similar results by Melaku et al. (2017) and Tsegaye, Vedeld and Moe (2013) also showed that occurrences of droughts had led to crop failure in the Afar Region. In discussion, district agricultural office administrators, extension workers and focus group participants also pointed out that 5–7 years ago, the agropastoral villages were engaged in construction of nursery sites for seedling multiplication of multipurpose trees (pasture, medicinal plants and edible fruits). However, in recent years, because of shortage of rainfall and drying up of private and communal water ponds, these activities were not successful. In addition, crop yields (for example, sorghum and maize) have been decreasing in the study area.

#### Livestock asset reduction

Livestock has been an important asset of the pastoralists and agropastoralist in the study area. The major livestock species reared by the respondents were sheep, goats, camels, cattle and donkeys. However, the recurring droughts along with increased heat stress affected livestock holdings per household. About 81% of agropastoralists and 100% of the pastoralists felt climate change reduced their livestock numbers and productivity. The key informant interview participants also complained that animal mortality has been increasing because of shortage of pasture and diseases, especially during drought times. Many livestock owners were altogether obliged to use the same water sources for their animals to drink, and concentration of livestock triggered the spread of disease. They indicated that tick infestation, anthrax, sheep and goat pox and pneumonia were common diseases observed during the drought and at the beginning of the rainfall after drought in the study area. This result was in line with Ayal and Leal Filho (2017), who reported that the rising temperatures and droughts caused the shortage of water and pasture for livestock as well as the spread of livestock diseases. Similarly, a study conducted by Akerlof, Maibach and Fitzgerald (2013) reported that in dry land areas, livestock are becoming weaker and underweight as a result of a steady decline in pasture and water availability, exposing them to infections during the dry season.

#### Food shortage and loss of income

According to the findings, recurrent droughts and unpredictable precipitation reduced crop and livestock production, resulting in increasing food insecurity and income loss. Food shortages were reported by the majority of agropastoral households (60.32%) and 97.5% of pastoralists because of livestock death and crop failure during droughts. Furthermore, climate-related shocks resulted in a loss of income for 81% of agropastoral households and 90% of pastoralists. This result corroborates the IPCC (2013) report, which stated that the impact of climate change in east Africa will increase the risk of food insecurity and the collapse of food systems, along with the risk of loss of rural livelihoods and income because of insufficient access to drinking and irrigation water, as well as decreased crop and livestock productivity, particularly for pastoralists with limited capital in arid and semi-arid areas. Similarly, Zenebe et al. (2011) found a decline in average incomes of rural farmers as a result of climate-related shocks, and Gebreegziabher et al. (2011) found a 30% loss of income in Ethiopian rural areas as a result of climate change and variability.

## Conclusions and recommendations

The findings of this study show that pastoral households in the study area perceived an increasing trend in annual temperature and a decreasing trend in annual and seasonal rainfall. Except for a long-term decrease in rainfall, Mann–Kendall’s trend analysis confirmed pastoral communities’ perceptions of increased temperatures and rainfall variability. The finding confirmed significant increasing trends of temperature in the study area. However, significant changes were not observed in the long-term annual and seasonal rainfall. Interseasonal and annual rainfall distribution was highly irregular. Moreover, the intraseasonal and annual rainfall was highly variable in the study area. The results revealed that six droughts (one severe drought and five moderate droughts) were observed for the period 1983–2017. Therefore, the meteorological impact in the study area could be highly associated with the increased temperature, recurrent droughts, irregular distribution of rainfall within a season and year and high rainfall variability amongst seasons and years. The results indicated that the perceived impacts of climate variability included reduced livestock asset, crop failure, water scarcity, food shortage and loss of income. Therefore, there is a need to provide farmers with disease-resistant and drought-resistant, early-maturing crops and livestock breeds. It is also paramount to build an early warning system before the occurrence of climate-related hazards such as drought, diseases and pests. Water-related interventions such as water harvesting during good rainy seasons and improving small-scale irrigation farming are paramount if pastoral households need to be climate-resilient. Enhancing pastoral communities’ access to education and awareness programmes will improve their understanding of climate change and their ability to implement environment-friendly adaption strategies.
